# Effect of MTHFR Polymorphisms on Gastrointestinal Cancer Risk in Italy

**DOI:** 10.14740/wjon930w

**Published:** 2015-08-27

**Authors:** Federica Mazzuca, Marina Borro, Andrea Botticelli, Laura Aimati, Giovanna Gentile, Carlo Capalbo, Chiara Maddalena, Eva Mazzotti, Maurizio Simmaco, Paolo Marchetti

**Affiliations:** a Department of Clinical Oncology, Faculty of Medicine and Psychology, University of Rome “La Sapienza”, Azienda Ospedaliera Sant’Andrea, Via di Grottarossa, 1035/39, 00189 Roma, Italy; b Department of Advanced Molecular Diagnosis Unit (DiMA), Azienda Ospedaliera Sant’Andrea, Via di Grottarossa, 1035/39, 00189 Roma, Italy

**Keywords:** MTHFR, Polymorphisms, Gastrointestinal, Cancer risk, Colorectal

## Abstract

**Background:**

The aim of the study was to assess the association of single nucleotide polymorphisms (SNPs) C677T and A1298C in the methylenetetrahydrofolate reductase gene with colorectal, esophageal/gastric and pancreatic cancer in a cohort of Italian patients.

**Methods:**

A total of 790 cancer patients and 202 healthy controls were genotyped and distributions in genotype and allele frequencies were compared by Chi-squared analysis and logistic regression analysis.

**Results:**

According to most of previous findings, we found an effect of the C677T variant, but no effect of the A1298C, in colorectal and esophageal/gastric, whereas no association was evidenced with pancreatic cancer. We found that only homozygous TT carriers of the C677T variant had an increased risk for onset of cancer.

**Conclusion:**

This result could be related to dietary and behavioral habits of the analyzed population, which could mitigate the deleterious effect of the T allele in heterozygosity and it highlights the importance to validate genetic determinant of cancer risk in different population and geographical areas.

## Introduction

Methylenetetrahydrofolate reductase (MTHFR) is an enzyme that catalyzes the conversion of 5,10-methylenetetrahydrofolate to 5-methyltetrahydrofolate, the primary circulatory form of folate, which is a cofactor for homocysteine-methionin conversion by methionine synthase [1, 2]. This step is crucial to maintain the metabolic function of folate, which plays a pivotal role in DNA synthesis, repair and methylation [3, 4].

MTHFR is a polymorphic gene, with the single nucleotide variants C677T in exon 4 (Ala to Val) and A1298C in exon 7 (Glu to Ala). Both variants are associated with a diminished specific activity of the enzyme [5, 6]. The C677T substitution unequivocally affects enzyme function and it has been associated with increased plasma homocysteine concentrations and an altered balance of folate metabolites, whereas the *in vivo* functional relevance of the A1298C variant is less well defined. Patients who are TT homozygous for C677T have been reported as having 30% of normal enzyme activity, while heterozygotes (CT) have been reported as having 65% of enzyme activity, compared with the wild-type gene variant (CC). The reduction of the MTHFR activity leads to a hypomethylation of DNA, one of the major events implicated in the carcinogenesis.

Several studies reported an involvement of the MTHFR C677T and A1298C polymorphisms in the etiology of colorectal, gastric and esophageal cancer, while the association with pancreatic cancer is still controversial [7, 8].

The identification of genetic factors affecting the gastrointestinal (GI) cancer risk would be useful to actuate an earlier intervention on modifiable risk factors as lifestyle and diet, thus improving preventive medicine [9, 10].

However, the contribution of gene polymorphisms to disease susceptibility can vary with ethnicity, lifestyle and environmental factor. Thus, to implement clinical use of genetic prediction factors, it is important to validate results from association studies in specific population and geographic region.

This study was aimed to evaluate the MTHFR genotype as a GI cancer risk parameter in the Italian population.

## Material and Methods

### Patients

The study included 790 cancer patients (403 males, 387 females, aged 27 - 87 years) followed at the Sant’Andrea Hospital of Rome, Italy, between April 2009 and April 2013, and 202 healthy controls (95 males, 107 females, aged 32 - 75 years).

Inclusion criteria for cancer patients were: age > 18 years; histologically documented GI cancer; Eastern Cooperative Oncology Group performance status 2 or less; written informed consent to collect and to use patient’s 5 mL blood. Exclusion criteria were relevant comorbidities within 6 months before the enrolment (i.e. myocardial infarction, lung fibrosis, etc.). Of the 790 recruited patients, 553 were affected by colorectal cancer, 138 by esophageal and gastric cancer, and 99 by pancreatic and biliar duct cancer (cancer subtypes shown in Table 1).

**Table 1:** Cancer Subtypes in the Patient’s Cohort

Cancer type	No. (%)
Colorectal cancer	553 (100)
Colon cancer	290 (52)
Rectal cancer	263 (48)
Esophageal/gastric cancer	138 (100)
Gastric cancer	123 (89)
Esophageal cancer	11 (8)
Junction E/G	4 (3)
Bilio-pancreatic cancer	99 (100)
Pancreatic cancer	74 (74.7)
Biliary duct cancer	25 (25.3)

All participants gave their informed consent prior to their inclusion in the study. The study was conducted in accordance with the Declaration of Helsinki, 1964, revised in 2004. The protocol was approved by the institutional ethic committee.

### Genotyping

Five milliliter of venous blood was collected with ethylene diamine tetra acetic acid (EDTA) as anticoagulant. Genomic DNA was extract from peripheral blood leukocytes using the Helix Extraction System (Diatech) and the X-Tractor Gene (Corbett Robotics).

MTHFR C677T (rs1801133) and A1298C (rs1801131) were genotyped in a single reaction using a multiplexed single-nucleotide extension system (IPlex chemistry, Sequenom, San Diego, CA, USA) following the manufacturer’s instruction. The extended products were analyzed using the MassArray Analyzer (Sequenom). Locus-specific amplification and extension primers were designed using the Assay Design Software (Sequenom). The primers’ sequences were for MTHFR C677T: 5’-ACGTTGGATGCTTGAAGGAGAAGGTGTCTG-3’ (forward), 5’-ACGTTGGATGTGCATGCCTTCACAAAGCGG-3’ (reverse), 5’-GCGTGATGATGAAATCG-3’ (extension). For MTHFR A1298C: 5’-ACGTTGGATGTCTCCCGAGAGGTAAAGAAC (forward), 5’-ACGTTGGATGAGGAGCTGCTGAAGATGTGG-3’ (reverse), 5’-GAGGAGCTGACCAGTGAAG-3’ (extension).

### Statistics

Test for deviation from the Hardy-Weinberg (HW) equilibrium, analysis of genotype and allele distributions were performed using the SNPStats software [11, 12]. The software uses logistic regression model, adjusting for age and sex, and different inheritance models to estimate the odds ratios (ORs) for each genotype respect to the reference genotype in the control group. The models of inheritance used are: co-dominant (assumes that every genotype gives a different and non-additive risk); dominant (assumes that a single copy of the mutant allele is enough to modify the risk, and then heterozygous and homozygous mutant genotypes have the same risk), recessive (assumes that two copies of the mutant allele are necessary to change the risk, and then the heterozygous and wild-type homozygous genotypes have the same effect) and log-additive (assumes that each copy of the mutant allele modifies the risk in an additive form, and then the mutant homozygous have double risk than heterozygous).

## Results

Distribution of genotype and allele frequencies of MTHFR C677T and A1298T polymorphisms in controls and patients, grouped by cancer type, is shown in Table 1.

No difference in frequencies of the MTHFR A1298C was detected, whereas the C677T polymorphisms was found to be associated with colorectal and gastric cancer, but not with pancreatic cancer. In particular, the TT genotype was found in 21.6% of the colorectal cancer cases and in 23.2% of the esophageal/gastric cancer cases compared to the 13.9% frequency in the control group. As shown by logistic regression analysis (Table 2), the TT genotype was associated with an increased risk of colorectal cancer ranging from the 1.38 OR using a log-additive model of inheritance to a 1.98 OR using the co-dominant model of inheritance. The TT genotype was also associated with an increased esophageal/gastric cancer risk ranging from the 1.52 OR using a log-additive model of inheritance to a 2.35 OR using the co-dominant model of inheritance (Table 3).

**Table 2:** Genotype and Allele Frequencies in the Analyzed Population (n = 991)

MTHFR polymorphism group	Genotype, n (%)			P*	Alleles, n (%)		P*
	CC	CT	TT		C	T	
C677T							
Control (n = 202)	76 (37.6)	98 (48.5)	28 (13.9)		250 (62.9)	154 (38.1)	
Colorectal cancer (n = 550)	163 (24.7)	268 (48.7)	119 (21.6)	0.023	594 (54.0)	506 (46.0)	0.06
Esophageal/gastric cancer (n = 138)	37 (26.8)	69 (50.0)	32 (23.2)	0.031	143 (51.8)	133 (48.2)	0.009
Pancreatic/biliar cancer (n = 97)	32 (33.0)	51 (52.6)	14 (14.4)	0.733	115 (59.3)	79 (40.7)	0.541
	AA	AT	TT		A	T	
A1298T							
Control (n = 202)	103 (51.0)	80 (39.6)	19 (9.4)		286 (70.8)	118 (29.2)	
Colorectal cancer (n = 553)	263 (47.5)	232 (41.9)	58 (10.6)	0.695	758 (68.5)	348 (31.5)	0.401
Esophageal/gastric cancer (n = 138)	60 (43.8)	66 (48.2)	11 (8.0)	0.298	186 (67.9)	88 (32.1)	0.419
Pancreatic/biliar cancer (n = 99)	49 (49.5)	45 (45.5)	5 (5.0)	0.340	143 (72.2)	55 (27.8)	0.716

*Difference in genotypes or alleles distribution between each cancer group and the control group assessed by X^2^ statistical testing.

**Table 3:** Single SNPs Association Analysis of MTHFR Polymorphism With Risk of Each Type of Cancer, According to Multiple Inheritance Models

MTHFR polymorphism	Model	Genotype	Colorectal cancer		Esophageal/gastric cancer		Pancreatic/biliar cancer	
			OR (95% CI)	P*	OR (95% CI)	P*	OR (95% CI)	P*
C677T	Co-dominant	CC	1.00	0.02	1.00	0.031	1.00	0.73
		CT	1.28 (0.89 - 1.82)		1.45 (0.88 - 2.38)		1.24 (0.72 - 2.11)	
		TT	1.98 (1.21 - 3.25)		2.35 (1.24 - 4.46)		1.19 (0.55 - 2.55)	
	Dominant	CC	1.00	0.039	1.00	0.036	1.00	0.43
		CT + TT	1.43 (1.02 - 2.01)		1.65 (1.03 - 2.64)		1.23 (0.74 - 2.04)	
	Recessive	CC + CT	1.00	0.014	1.00	0.028	1.00	0.89
		TT	1.72 (1.10 - 2.68)		1.88 (1.07 - 3.29)		1.05 (0.52 - 2.10)	
	Log-additive	-	1.38 (1.09 - 1.74)	0.0064	1.52 (1.11 - 2.09)	0.0087	1.12 (0.78 - 1.61)	0.53
A1298C	Co-dominant	AA	1.00	0.7	1.00	0.29	1.00	0.32
		AT	1.14 (0.81 - 1.60)		1.42 (0.90 - 2.23)		1.18 (0.72 - 1.95)	
		TT	1.20 (0.68 - 2.11)		0.99 (0.44 - 2.23)		0.55 (0.20 - 1.57)	
	Dominant	AA	1.00	0.4	1.00	0.19	1.00	0.81
		AT + TT	1.15 (0.83 - 1.58)		1.34 (0.86 - 2.06)		1.06 (0.66 - 1.72)	
	Recessive	AA + AT	1.00	0.66	1.00	0.66	1.00	0.17
		TT	1.13 (0.65 - 1.95)		1.42 (0.91 - 2.20)		0.51 (0.19 - 1.42)	
	Log-additive	-	1.11 (0.87 - 1.42)	0.41	1.15 (0.82 - 1.61)	0.42	0.93 (0.64 - 1.36)	0.71

*By logistic regression model adjusted for age and sex.

## Discussion

Several studies suggested that the C677T and A1298C polymorphisms in the MTHFR gene play an important role in GI cancer risk. The mechanism underlying this observation is related to the impairment of MTHFR activity due to the presence of variant alleles, leading to an alteration of the folate metabolism, which is implied in DNA synthesis, replication and regulation by methylation.

Even if several recent studies and meta-analysis reported association between these MTHFR polymorphisms and GI cancer risk [7], no consensus has been reached concerning the utility of these genetic markers as risk predictors. It is noteworthy to consider that folate metabolism, and its role in carcinogenesis, are affected by key non-genetic factors, as diet (folate intake) or alcohol consumption [13, 14]. Thus, the utility of genetic markers as cancer risk predictors may vary according to ethnical distribution of variant alleles and to specific gene-environment interaction in different population/geographical area. This view has been also stressed by Xia et al commenting results from a meta-analysis that evaluated the variation of MTHFR C677T and gastric cancer risk [15]. Few small and heterogeneous association studies investigated the role of MTHFR polymorphisms and cancer risk in an Italian population. We genotyped a cohort of 790 patients with GI cancer and a cohort of 202 non-cancer subjects. In this sample, the A1298C polymorphism was unrelated with GI cancer development, confirming most of previous results.

The homozygous carriers of the C677T polymorphisms presented a 1.98 OR increased risk to develop colorectal cancer and 2.35 OR increased risk to develop esophageal/gastric cancer, but no increased risk to develop pancreatic cancer. This result confirms earlier studies reporting an effect of C677T upon colorectal cancer risk and esophageal/gastric cancer risk. However, the recent meta-analysis [15] reported that both heterozygous and homozygous carriers of the variant allele (CT and TT) had a higher risk of developing gastric cancer, whereas in the Italian population, we found increased risk only in the homozygous TT carriers. This difference may be explained by a balancing effect of the Mediterranean diet in heterozygous, which has a higher activity of MTHFR compared to homozygous.

Concerning the bilio-pancreatic cancer, the lack of association of the C677T polymorphism and increased cancer risk agrees with the meta-analysis of Liu et al [8].

Limits of this study are represented by the low number of patients in the pancreatic cancer subgroup and by the lack of information regarding diet, folate intake and alcohol consumption. However, the studied cohort included individuals from the same region which are supposed to have similar diets and habits.

In conclusion, this report highlights that in the Italian population, only the TT genotype of the MTHFR C677T polymorphisms is associated with colorectal and esophageal/gastric cancer and should be considered as an additional risk factor for these cancer types, functional to the implementation of earlier and more targeted preventive strategies.
